# Granulomatous rosacea: a case report 

**DOI:** 10.1186/s13256-017-1401-5

**Published:** 2017-08-20

**Authors:** A. Kelati, F. Z. Mernissi

**Affiliations:** Department of Dermatology, University Hospital Hassan II, 202 Hay Mohamadi, Fez, Morocco

**Keywords:** Granulomatous rosacea, Rare clinical form, Dermoscopy, Histology, Case report

## Abstract

**Background:**

Granulomatous rosacea is a rare chronic inflammatory skin disease with an unknown origin. The role of *Demodex follicularum* in its pathogenesis is currently proved.

**Case presentation:**

We report a case of a 54-year-old Moroccan man with a 3-month history of erythematous, nonpruritic papules on the lateral side around the eyes. Dermoscopy and histology confirmed the diagnosis of granulomatous rosacea.

**Conclusions:**

We describe another clinical presentation of granulomatous rosacea with a clinical-dermoscopic-pathological correlation.

## Background

Granulomatous rosacea (GR) is a rare chronic inflammatory skin disease reported primarily in middle-aged women [1]. It is thought to be a particular form of rosacea on the basis of unique clinical and histological findings of granulomas; it is characterized by erythematous papules most commonly affecting the face; and it tends to have a chronic course [2]. We present the case of a 54-year-old man with a 3-month history of GR associated with demodecidosis.

## Case presentation

**Fig. 1:**
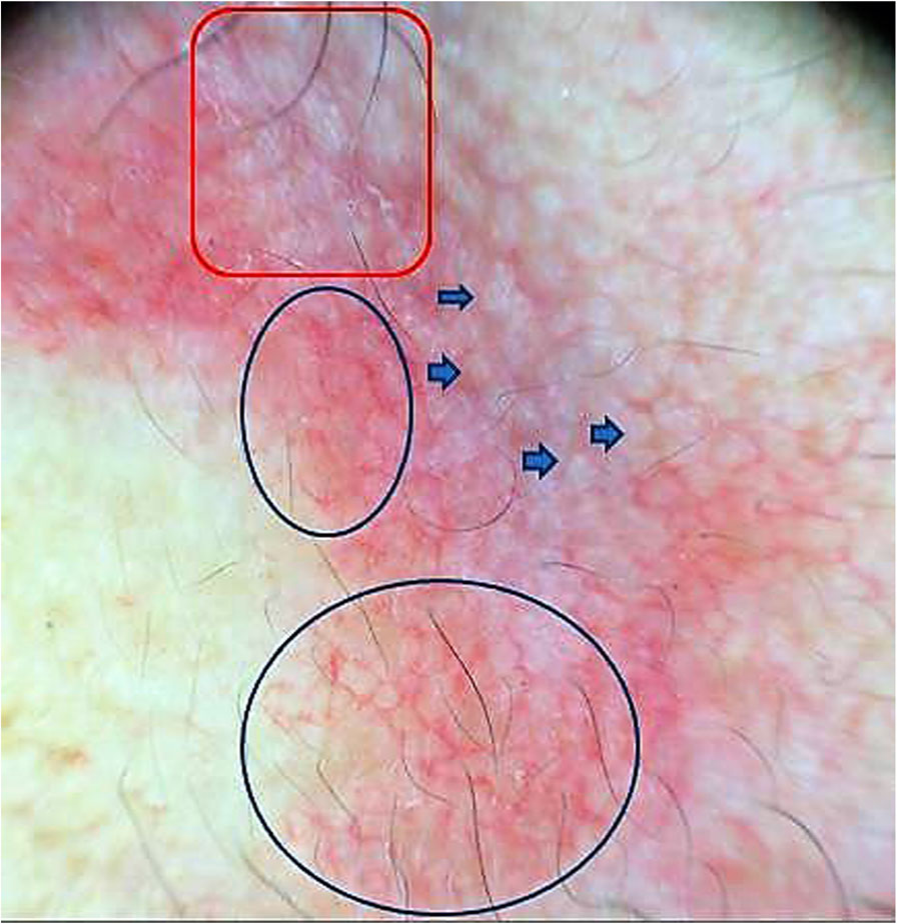
Granulomatous rosacea. Dermoscopy reveals follicular openings containing white grayish plugs (*Demodex* follicular openings) (*blue arrows*), creamy whitish linear structures filling the follicular openings (*Demodex* tails) (*red rectangle*), and polygonal vessels (*blue circles*)

A 54-year-old Moroccan man with a history of psoriasis in remission presented with a 3-month history of erythematous nonpruritic lesions of the face with hypersensitivity to heat. A clinical examination revealed erythematous, telangiectatic, confluent papules of the lateral side around the eyes without any scales, crusts, or pustules. In the differential diagnosis, we included GR, sarcoidosis, lupus vulgaris, and lupus erythematosus tumidus. Dermoscopy revealed linear vessels characteristically arranged in a polygonal network, creamy and whitish linear areas, and white grayish plugs surrounded by an erythematous halo filling the follicular openings (Fig. 1).

**Fig. 2:**
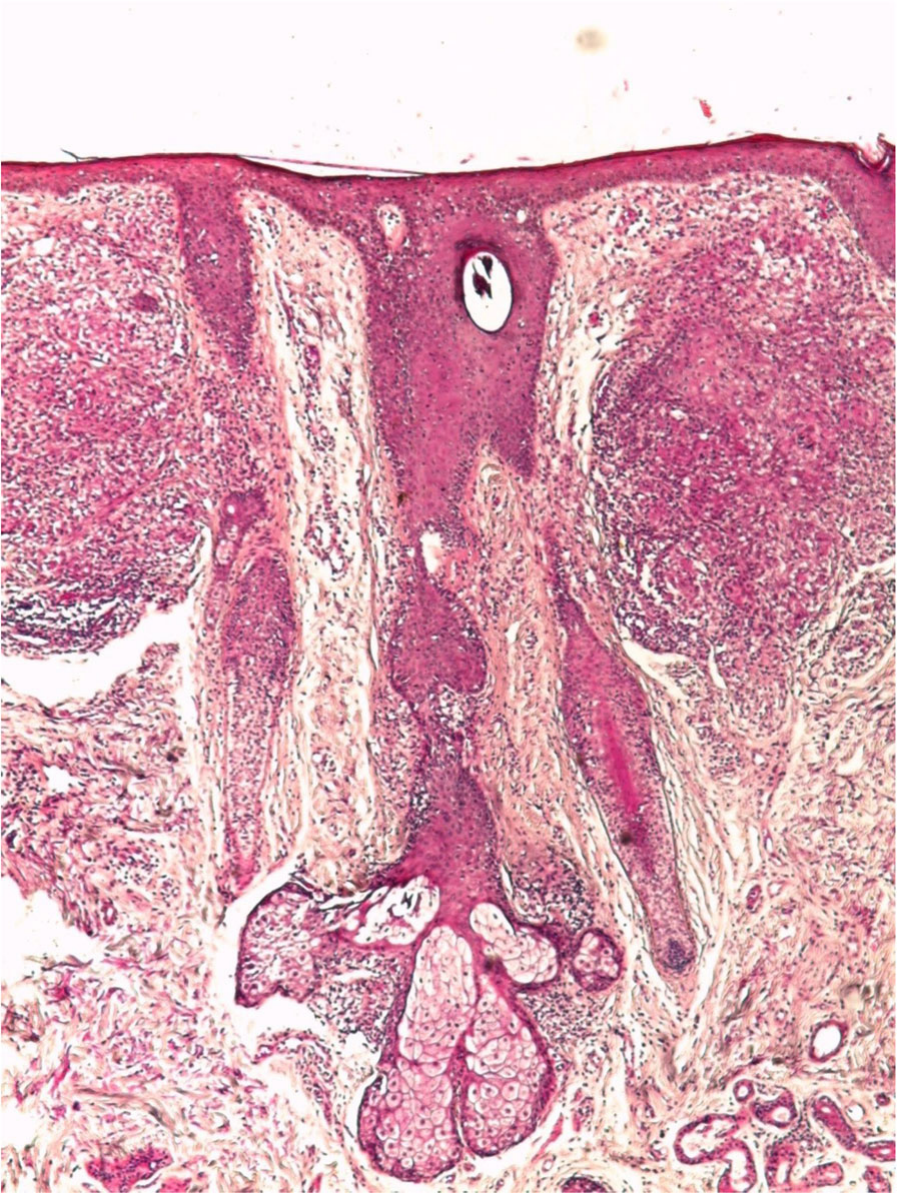
Granulomatous rosacea. Histological findings (hematoxylin and eosin stain, original magnification × 10) were granulomatous inflammatory infiltrates destroying the hair follicles with the presence of *Demodex folliculorum* at the follicular ostium

Histological examination of a punch biopsy specimen of the lesion showed granulomatous dermatitis with the presence of *Demodex folliculorum* on the biopsied tissue. These clinical, dermoscopic, and histological findings were consistent with the diagnosis of GR (Fig. 2), and the patient was treated with topical metronidazole for a total of 10 weeks, which led to a significant improvement.

## Discussion

The role of *Demodex* in GR’s pathogenesis was debated in the last decade, with recent data highlighting its significant role in triggering GR [3]. Our observation confirmed that this mite plays an important role in stimulation of the immune system and the formation of granulomas in GR in unusual sites. In our patient, the presence of *Demodex* in the follicular openings was confirmed by histology, and we noticed that a granulomatous infiltrate was agglomerated around the pilosebaceous follicles containing the mite. Dermoscopy confirms the presence of the characteristic vascular polygons, not only in conventional rosacea [4, 5] but also in GR. In our patient, we also noticed the most indicative dermoscopic whitish features of demodicidosis, called *Demodex tails* and *Demodex follicular openings* [6, 7].

## Conclusions

We report an original observation of GR with a rare localization and an association with demodicidosis.
